# Lymphatic endothelial cells efferent to inflamed joints produce iNOS and inhibit lymphatic vessel contraction and drainage in TNF-induced arthritis in mice

**DOI:** 10.1186/s13075-016-0963-8

**Published:** 2016-03-12

**Authors:** Qianqian Liang, Yawen Ju, Yan Chen, Wensheng Wang, Jinlong Li, Li Zhang, Hao Xu, Ronald W. Wood, Edward. M. Schwarz, Brendan F. Boyce, Yongjun Wang, Lianping Xing

**Affiliations:** Department of Orthopaedics, Longhua Hospital, Shanghai University of Traditional Chinese Medicine, 725 South Wanping Road, Shanghai, 200032 China; Department of Pathology and Laboratory Medicine, University of Rochester Medical Center, 601 Elmwood Avenue, Rochester, NY 14642 USA; Center for Musculoskeletal Research, University of Rochester Medical Center, 601 Elmwood Avenue, Rochester, NY 14642 USA; Departments of Obstetrics and Gynecology, University of Rochester Medical Center, 601 Elmwood Avenue, Rochester, NY 14642 USA; Institute of Spine, Shanghai University of Traditional Chinese Medicine, 725 Wan-Ping South Road, Shanghai, 200032 China

**Keywords:** Rheumatoid arthritis, TNF, Lymphatic function, Lymphatic endothelial cells (LECs), Lymphatic smooth muscle cells (LSMC), Inducible nitric oxide synthase (iNOS), Ferulic acid

## Abstract

**Background:**

In this study, we sought to determine the cellular source of inducible nitric oxide synthase (iNOS) induced in lymphatic endothelial cells (LECs) in response to tumor necrosis factor (TNF), the effects of iNOS on lymphatic smooth muscle cell (LSMC) function and on the development of arthritis in TNF-transgenic (TNF-Tg) mice, and whether iNOS inhibitors improve lymphatic function and reduce joint destruction in inflammatory erosive arthritis.

**Methods:**

We used quantitative polymerase chain reactions, immunohistochemistry, histology, and near-infrared imaging to examine (1) iNOS expression in podoplanin + LECs and lymphatic vessels from wild-type (WT) and TNF-Tg mice, (2) iNOS induction by TNF in WT LECs, (3) the effects of iNOS inhibitors on expression of functional muscle genes in LSMCs, and (4) the effects of iNOS inhibitors on lymphatic vessel contraction and drainage, as well as the severity of arthritis, in TNF-Tg mice.

**Results:**

LECs from TNF-Tg mice had eight fold higher *iNOS* messenger RNA levels than WT cells, and iNOS expression was confirmed immunohistochemically in podoplanin + LECs in lymphatic vessels from inflamed joints. TNF (0.1 ng/ml) increased *iNOS* levels 40-fold in LECs. LSMCs cocultured with LECs pretreated with TNF had reduced expression of functional muscle genes. This reduction was prevented by ferulic acid, which blocked nitric oxide production. Local injection of L-N^6^-(1-iminoethyl)lysine 5-tetrazole-amide into inflamed paws of TNF-Tg mice resulted in recovery of lymphatic vessel contractions and drainage. Treatment of TNF-Tg mice with ferulic acid reduced synovial inflammation as well as cartilage and bone erosion, and it also restored lymphatic contraction and drainage.

**Conclusions:**

iNOS is produced primarily by LECs in lymphatic vessel efferent from inflamed joints of TNF-Tg mice in response to TNF and inhibits LSMC contraction and lymph drainage. Ferulic acid represents a potential new therapy to restore lymphatic function and thus improve inflammatory arthritis by inhibiting local production of nitric oxide by LSMCs.

**Electronic supplementary material:**

The online version of this article (doi:10.1186/s13075-016-0963-8) contains supplementary material, which is available to authorized users.

## Background

The lymphatic system plays a critical role in maintenance of fluid homeostasis and normal immune responses [1]. It is also an important modulator of pathological processes, such as inflammation, because it removes harmful factors and cells from disease sites [2]. The importance of lymph drainage in rheumatoid arthritis (RA) has been established in studies demonstrating that (1) lymph efferent from inflamed joints contains high levels of cytokines and chemokines [3], (2) synovial specimens from patients with RA and mice with inflammatory erosive arthritis have increased numbers of lymphatic vessels [4–6], and (3) the size of draining lymph nodes is a biomarker of arthritic flare [7–9]. Additional preclinical studies using the tumor necrosis factor–transgenic (TNF-Tg) [10] and K/B × N [11] mouse models of chronic inflammatory erosive arthritis have also revealed defects in lymphatic drainage in these models (recently reviewed [12]). In summary, longitudinal in vivo imaging and cell labeling studies have demonstrated that arthritis progression commences with decreased lymphatic clearance [8, 13], which is associated with dysfunction of collecting lymphatic vessels draining affected joints, including reduced or absent contractility [14] and decreased pumping pressure [15]. However, the cellular and molecular mechanisms responsible for impaired lymphatic function during arthritis progression are not fully understood.

The lymphatic vasculature is composed of capillaries and collecting vessels that have different morphology, structural composition, and function [1]. For example, lymphatic capillaries consist of a single layer of lymphatic endothelial cells (LECs), which are phenotypically characterized by surface expression of lymphatic vessel endothelial hyaluronan receptor 1 (LYVE-1), podoplanin (PDPN), or CD31 [2]. Collecting lymphatic vessels have valves that maintain unidirectional flow and are covered by lymphatic smooth muscle cells (LSMCs) that express α-smooth muscle actin (SMα) [16, 17]. Unlike the cardiovascular system, the lymphatic system has no central pump. Thus, lymph movement is achieved by alternating contraction and relaxation of LSMCs that propels lymph through valves to upstream draining lymph nodes and eventually to the venous circulation [18, 19]. Mechanical compression during contractions of adjacent skeletal muscle and changes in pressure in arteries also promote lymph transport along the collecting vessels [18, 19]. Therefore, an emerging theory of impaired function in lymphatic vessel efferent from arthritic joints during chronic systemic inflammation in RA is that there is a breakdown in the function of their LECs and/or LSMCs that results in afferent synovitis due to decreased lymph egress from affected joints [9, 12]. Initially, this breakdown involves loss of efferent lymphatic vessel contractions via undefined mechanisms, which leads to LEC and LSMC damage, apoptosis, and extensive lymphatic vessel destruction by inflammatory cells that become resident due to the absence of lymph flow [12]. Thus, elucidating the mechanisms responsible for the loss of lymphatic contractions and developing interventions to restore efferent lymph flow from arthritic joints are important frontiers in RA research and drug development.

Nitric oxide (NO) is a primary regulator of lymphatic vessel contraction via its vasodilatory signal in LSMCs [20, 21]. In a mouse model of acute inflammation, contraction of LSMCs is attenuated by excessive NO produced by inducible nitric oxide synthase (iNOS)-expressing CD11b^+^Gr-1^+^ myeloid cells [22]. Most recently, Scallan and Davis used an elegant ex vivo model to demonstrate that genetic removal of basal NO enhances contractile activity in isolated murine collecting lymphatic vessels [23]. Several studies have also demonstrated NO and iNOS involvement in inflammatory arthritis, including increased circulating NO levels in serum, urine, and synovial fluid of patients with RA [24, 25], as well as higher iNOS levels in synovial tissues of RA joints than in osteoarthritic joints and absence of iNOS in normal joints [26–29]. In addition, LECs can be induced with lipopolysaccharide in culture to express iNOS [30], NOS inhibitors reduce synovial inflammation and tissue damage in arthritic rats [31], and increased NO production in LECs causes impairment of lymphatic drainage in cirrhotic rats [32]. However, the factors regulating iNOS expression in lymphatic vessels efferent from inflamed joints and the effects of iNOS on lymph drainage during the development of inflammatory erosive arthritis have not been investigated. In the present study, we used TNF-Tg mice to identify the cellular source of iNOS expression in efferent lymphatic vessels from inflamed joints, the role of LEC-produced iNOS in inhibition of LSMC function, and the effects of NO inhibitors (*N*_ω_-nitro-l-arginine methyl ester [l-NAME] and l-N^6^-(1-iminoethyl)lysine 5-tetrazole-amide [l-NIL]), as well as ferulic acid (FLA; the active component of an herbal therapy for RA [33, 34]), on LEC-mediated LSMC inhibition, lymphatic dysfunction, and joint pathology. We provide the first evidence that LECs produce iNOS and that the resulting NO inhibits LSMC function. Furthermore, selective iNOS inhibitors restore LSMC function and lymph flow from affected joints and prevent inflammatory erosive arthritis in mice.

## Methods

### Animals

All murine studies were performed according to protocols approved by the University of Rochester Committee on Animal Resources. The 3647 line of TNF-Tg mice was generated by Dr. George Kollias (Institute of Immunology, Alexander Fleming Biomedical Sciences Research Center, Vari, Greece) [10]. The TNF-Tg mice were bred as heterozygotes on a C57BL/6 background, and their wild-type (WT) littermates were used as healthy controls. These TNF-Tg mice have normal ankle joints at age 1 month, display mild ankle joint inflammation and bone erosion at 2–2.5 months of age, and have severe erosive arthritis at >5 months of age [35, 36]. Primary LSMCs were generated from 1- to 2-month-old Sprague-Dawley rats that were purchased from Shanghai Laboratory Animal Center (Shanghai, China). These studies were conducted in adherence to the Guiding Principles for the Care and Use of Laboratory Animals according to the Regulations of the People’s Republic of China for Administration of Laboratory Animals. In vivo drug treatments were performed as follows. To evaluate the immediate effects of the iNOS inhibitor L-NIL (CAS 159190-45-1; Cayman Chemical, Ann Arbor, MI, USA), mice were anesthetized with approximately 2 % vol/vol isoflurane in oxygen, and indocyanine green (ICG) was injected into their footpads and visualized using near-infrared (NIR) imaging. Thirty minutes later, 0.1 ml (4 mg/kg) of l-NIL or saline was injected into footpads during the NIR-ICG imaging session. The lymphatic pulse and clearance were monitored by NIR-ICG imaging after treatment. The effects of long-term NO inhibition were assessed by adding 100 ng/ml of L-NIL or 100 ng/ml of the general NOS inhibitor L-NAME (CAS 51298-62-5; Cayman Chemical) to the drinking water, which was changed every day for 6 weeks. Ferulic acid (CAS 1135-24-6; Shanghai Forever Biotech Co., Ltd, Shanghai, China) was dissolved in saline. Mice were gavaged daily with ferulic acid solution (20 mg/kg/day) or saline for 12 weeks. We determined that the purity of ferulic acid was more than 98 % by high-performance liquid chromatography (Additional file 1).

### Immunohistochemistry

Immunohistochemistry (IHC) was performed on frozen sections of fresh popliteal lymph nodes (PLNs) and limb tissues containing lymphatic vessels obtained from the mice, as described previously [37]. Tissues were frozen in Tissue-Tek® O.C.T compound (Sakura Finetek, Torrance, CA, USA). To assist with identification of lymphatic vessels between the paws and PLNs, 0.5 % Evans Blue dye was injected into footpads 5 minutes before the animals were killed and tissues were dissected. Sections (6 μm thick) were cut from blocks containing PLNs and lymphatic vessels using the CryoJane Tape Transfer System (Leica Biosystems, Wetzlar, Germany). Before staining, slides were exposed to room temperature (RT) for at least 30 minutes. The sections were fixed in 4 % paraformaldehyde for 10 minutes at RT or in acetone/ethanol for 5 minutes at −20 °C, followed by washing with phosphate-buffered saline (PBS) three times for 5 minutes each. A blocking buffer (3 % bovine serum albumin [BSA] in PBS) was applied for 30 minutes at RT. Fluorescence-conjugated antibodies, including mouse anti-LYVE-1 Alexa Fluor 488 (eBioscience, San Diego, CA, USA), mouse anti-iNOS/NOS type II fluorescein isothiocyanate (FITC; BD Biosciences, San Jose, CA, USA), antimouse Gr-1 phycoerythrin (PE) (eBioscience), and hamster antimouse PDPN (Abcam, Cambridge, MA, USA), were diluted in blocking buffer and incubated with sections overnight at 4 °C. After being washed with 0.1 % PBS with Tween 20 three times, the sections were mounted with Molecular Probes ProLong Gold antifade reagent and 4′, 6-diamidino-2-phenylindole (DAPI; Life Technologies, Carlsbad, CA, USA). Images were taken under a Zeiss Axio Imager (Carl Zeiss Microscopy, Oberkochen, Germany) and analyzed by AxioVision Rel (Carl Zeiss Microscopy).

### Flow cytometry and purification of synovial lymphatic endothelial cells

Soft tissue and muscles were removed from entire legs and digested with 2 ml of 1 mg/ml collagenase I (Worthington Biochemical, Lakewood, NJ, USA) for 1 h at 37 °C. Digestion was terminated by adding 8 ml of 2 % fetal bovine serum (FBS) in PBS and filtered with a 40-μm Falcon cell strainer (Corning, Corning, NY, USA). Centrifuged cells were washed twice with MACS buffer (0.5 % BSA, 2 mM ethylenediaminetetraacetic acid [EDTA] in PBS, pH 7.2), recentrifuged, and resuspended with 95 μl of MACS buffer plus 5 μl of PE-conjugated anti-PDPN antibody (catalog number 127407, lot B164982; BioLegend, San Diego, CA, USA) for 30 minutes at 4 °C, and then with 95 μl of MACS buffer plus 5 μl of anti-PE microbeads (catalog number 130-048-801; Miltenyi Biotec, Auburn, CA, USA) for another 30 minutes at 4 °C. Cells were resuspended with 0.5 ml of MACS buffer and passed through an LS separation column (catalog number 130-42-401; Miltenyi Biotec). After extensive washing, PDPN^+^ cells were collected from the LS column by flushing with 0.5 ml of MACS buffer. About 20,000 cells were obtained per mouse leg. To characterize PDPN^+^ cells as LECs, cells were stained with goat antimouse vascular endothelial growth factor receptor 3 antibody (catalog number AF743, lot DAB0231021; R&D Systems, Minneapolis, MN, USA) followed by Alexa Fluor 488–conjugated antigoat immunoglobulin G secondary antibody (catalog number A11078, lot 1613914; Life Technologies), or with biotin-conjugated mouse LYVE-1 (catalog number 13-0443-80; eBioscience) followed by allophycocyanin-conjugated streptavidin (catalog number 17-4317-82, lot E07261-169; eBioscience), or PE-conjugated mouse PDPN antibody, and subjected to flow cytometry (BD LSR II 12-color flow cytometer; BD Biosciences). The data were analyzed with FlowJo software (version 7.6.5; FlowJo, Ashland, OR, USA).

### Real-time polymerase chain reaction

RNA was extracted from LECs and LSMCs using TRIzol reagent (Life Technologies), and complementary DNA was prepared from total RNA using the GeneAmp RNA PCR Core Kit (Applied Biosystems, Foster City, CA, USA). Quantitative polymerase chain reaction (PCR) amplification was performed in triplicate with gene-specific primers and iQ SYBR Green Supermix (both from Bio-Rad Laboratories, Hercules, CA, USA) in an iCycler real-time PCR machine (Bio-Rad Laboratories). The relative abundance of each gene was calculated by subtracting the threshold cycle (C_t_) value of each sample for an individual gene from the corresponding C_t_ value of *β-actin* (ΔC_t_). The ΔΔC_t_ value was obtained by subtracting the ΔC_t_ of the test samples from the ΔC_t_ of control samples. These values were then raised to the power of 2 (2^−ΔΔCt^) to yield the fold expression relative to the reference level. The expression levels of functional muscle genes were examined using sequence-specific primers, which are listed in Additional file 2.

### Western blot analysis

Whole-cell lysate samples were fractionated by sodium dodecyl sulfate–polyacrylamide gel electrophoresis and transferred to nitrocellulose membranes (20 μg of protein/lane). Immunoblotting was carried out using antibodies to smooth muscle myosin heavy chain 2 ([sMYH2] catalog number ab53219, lot GR162408-3; Abcam), h1-calponin ([h1-Cal] catalog number C2687, lot 052 M4849; Sigma-Aldrich, St. Louis, MO, USA), SMα22) (catalog number ab14106, lot SR103317-2; Abcam), nuclear factor κB2 ([NF-κB2] catalog number 8242, lot 1; Cell Signaling Technology, Danvers, MA, USA) at 1:1000 dilution, iNOS (catalog number ab3523, lot GR187485-2; Abcam) at 1:800 dilution, and β-actin (catalog number A2228, lot 052M4816V; Sigma-Aldrich) at 1:5000 dilution. Bands were visualized using enhanced chemiluminescence (Amersham, Little Chalfont, UK). Signal intensity of bands was measured by densitometry.

### Nitric oxide levels

LECs were seeded at 10^6^/well in six-well plates overnight, pretreated with 1 μM aminoguanidine hemisulfate salt (Ami) and FLA (250 μM) for 3 h, and then with 1 ng/ml TNF for 24 h. Supernatants were collected, and nitrite levels were assessed using an NO assay kit (catalog number A012; Nanjing Jian Cheng Bioengineering Institute, Nanjing, China). This kit transforms nitrate to nitrite via nitrate reductase first, and then uses Griess reagent to detect the total amount of nitrite, from which the total NO levels are calculated. It does not detect reactive oxygen species [38].

### Isolation of lymphatic smooth muscle cells

We followed a published protocol for isolating LSMCs [39]. Mesenteric lymphatic vessels from 1- to 2-month-old rats were identified by injecting 10 μl of 0.5 % Evans blue dye into the mesenteric lymph nodes. Blue-stained lymphatic vessels that were easily separated from blood vessels were harvested, cut into small pieces, and transferred to a 1 % gelatin-coated plastic tissue culture dish. We used rats for these isolations because it is impossible to isolate LSMCs from mouse mesenteric lymphatics, owing to their much smaller size and difficulty in separating lymphatic from blood regular vessels. Cells were cultured in high-glucose Dulbecco’s modified Eagle’s medium supplemented with 20 % FBS, 2 mM sodium pyruvate, 2 mM l-glutamine, and antibiotics. The culture medium was changed every 3 days. Smooth muscle cells covering lymphatic vessels migrated from the vessels after 3–4 days, and vessel segments were removed aseptically. The cells were trypsinized after 7–10 days with 0.25 % trypsin in 0.02 % EDTA and transferred to a new gel-coated dish (passage 0). After 1–2 weeks, the cells reached confluence and were split into two dishes (passage 1). The cells were fixed with 10 % formalin for 10 minutes, blocked with 0.2 % Triton-100 and 1 % BSA in PBS for 30 minutes at RT, stained with FITC anti-αSMA antibody (1:400) at 4 °C overnight, and observed under a fluorescence microscope (Olympus IX 71; Olympus America, Center Valley, PA, USA). Whole-cell lysates were examined by Western blot analysis using a 1:1000 dilution of an anti-sMYH antibody (catalog number ab53219, lot GR162408-3; Abcam) and a 1:5000 dilution of anti-β-actin antibody (catalog number A2228, lot 052M4816V; Sigma-Aldrich).

### Coculture of LECs and LSMCs

A murine LEC cell line established from Freund’s adjuvant–induced benign lymphangiomas [40] (cells provided by Dr. S. Ran from the University of Illinois, Springfield, Illinois, USA) was seeded on ten coverslips (15 mm × 0.25 mm thick) at 10^6^ cells/coverslip in a 10-cm dish. After 2 days, LECs were treated with 1 ng/ml TNF (catalog number 510-RT-010; R&D Systems) with or without a selective iNOS inhibitor (1 μM Ami, catalog number M7033; Sigma-Aldrich) for 24 h. Coverslips with LECs were then transferred into another 10-cm dish, which had already been coated with 2 × 10^5^ LSMCs for 4 days. After 24 h of coculture, coverslips with LECs were removed and LSMCs were harvested for analysis.

### Near-infrared indocyanine green lymphatic imaging

NIR-ICG lymphatic imaging was performed as described previously [13, 37]. In brief, fur was removed from mouse legs with a depilatory cream (Veet; Reckitt Benckiser, Slough, UK). A 6-μl solution of ICG in distilled water (0.1 mg/ml) was injected intradermally into footpads. As shown in Figs. 3 and 4, ICG signaling was measured using a custom-made NIR-ICG system in which the ICG fluorescence is excited with light from a tungsten halogen bulb with a nondichroic, parabolic reflector to enhance NIR output and passed through a narrow excitation filter (part number ICG-B; Semrock, Rochester, NY, USA). As shown in Fig. 5, the ICG signal was measured using a Fluobeam 800 system (Fluoptics, Grenoble, France) that is composed of an electrical box containing the laser, laser power supply, an analogical/numerical module and light-emitting diode (LED) power supply, and an optical head containing the charge-coupled device camera and the LED lamps. The imaging system was controlled using Fluobeam software (V3.0; Fluoptics). NIR fluorescence of the entire leg was recorded for 1 h to observe efferent collecting lymphatic vessels from the ankle, including joints, synovium, and surrounding soft tissue. Recording sessions were repeated at 6 and 24 h for comparison with recordings from the first 5 minutes after injection. Using ImageJ software (National Institutes of Health, Bethesda, MD, USA), regions of interest (ROIs) were identified in the footpad and collecting lymphatic vessels to estimate (1) percentage clearance from the footpad ROI at 1 h or 6 h and 24 h after ICG injection and (2) vessel contraction rate, based on events exceeding an ROI fluorescence intensity threshold 400 seconds after injection, as we described previously [13, 37].

### Histologic and histomorphometric analyses

Ankle joints were fixed in 10 % phosphate-buffered formalin, decalcified in 10 % EDTA for 21 days, and embedded in paraffin. A series of 4-μm-thick sections was cut, and a total of ten sections were collected and divided into three levels. Each level was 40 μm from the previous level. One section from each of the three levels was stained with Alcian Blue/Orange G or for tartrate-resistant acid phosphatase (TRAP) activity to identify osteoclasts. Sections of the entire ankle joint, including the distal tibia and proximal metatarsal bones, were digitized using an Olympus VS120-S5-E whole-slide imaging system (Olympus America). All images were analyzed using Olympus OlyVIA software (Olympus America). We outlined ROIs, which included distal tibia, talus, calcaneus, proximal metatarsal bone, synovial capsules, ligaments, and adjacent soft tissues. The total tissue area of the ROI was determined automatically with Olympus VS120 software and ranged from 3.77 mm^2^ to 4.89 mm^2^. The area of inflammation and bone area were expressed as a percentage of the total tissue area. The area of TRAP^+^ cells and cartilage area were expressed in square millimeters. The data are presented as the means from three levels.

### Statistical analysis

Data are presented as means ± standard deviations. Statistical analyses were performed with SPSS 16.0 software (SPSS, Chicago, IL, USA). Differences between two groups were compared using Student’s *t* test. One-way analysis of variance test followed by a Bonferroni-corrected posttest was used for comparisons of more than two groups. *p* Values <0.05 were considered statistically significant.

## Results

### iNOS induced by TNF in LECs results in NO inhibition of LSMC gene expression

**Fig. 1:**
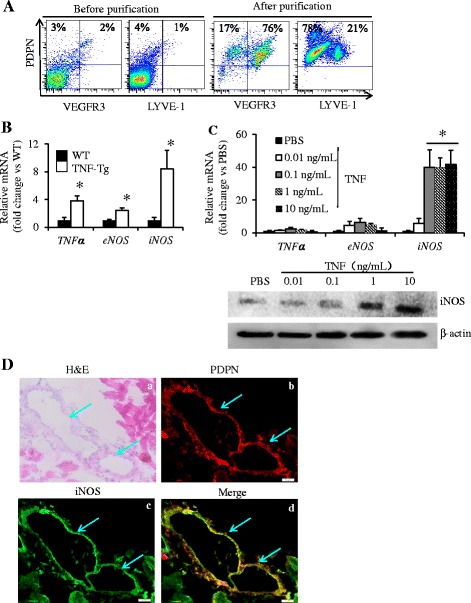
Lymphatic endothelial cells (LECs) in efferent vessels from arthritic paws of tumor necrosis factor–transgenic (TNF-Tg) mice express high levels of inducible nitric oxide synthase (iNOS). **a** Podoplanin-positive (PDPN^+^) LECs were isolated from joints of 7-month-old TNF-Tg mice and their wild-type (WT) littermates using phycoerythrin (PE) anti-PDPN antibody/anti-PE microbeads passed through an LS column. Cells were subjected to flow cytometry to assess their surface expression of LEC markers. **b** The expression levels of *TNF*, endothelial nitric oxide synthase (*eNOS*), and *iNOS* in LECs were determined by quantitative polymerase chain reaction (qPCR). The fold changes normalized to actin were calculated using a WT sample value as 1. Values are mean ± SD of five mice. **c** A murine LEC line was treated with different concentrations of TNF for 24 h. The expression levels of *TNF*, *eNOS*, and *iNOS* were determined by qPCR. The fold changes were calculated using phosphate-buffered saline (PBS)-treated sample values as 1. Values are mean ± SD of three samples. *p < 0.05 vs. PBS-treated samples. The expression levels of iNOS protein were determined by Western blot analysis. **d** Collecting lymphatic vessels and surrounding tissues were harvested from TNF-Tg mice older than 5 months of age. Frozen sections were stained with hematoxylin and eosin (H&E) or double–immunofluorescence-stained with anti-PDPN for LECs and anti-iNOS antibodies. H&E-stained section (*a*) shows a lymphatic vessel with an open lumen (indicated by *blue arrows*). Immunohistochemically stained sections show (*b*) PDPN^+^ lymphatic vessel endothelium (*red*), (*c*) iNOS^+^ vessels (*green*), and (*d*) merged PDPN^+^/iNOS^+^ LECs (*yellow*). Bar represents 200 μm. *mRNA* messenger RNA, *LYVE-1* lymphatic vessel endothelial hyaluronan receptor 1, *VEGFR3* vascular endothelial growth factor receptor 3

To define the cellular source of iNOS in efferent lymphatic vessels from affected joints in TNF-Tg mice, we performed multicolor fluorescence IHC on PLNs and lymphatic vessels draining the ankle [41]. Consistent with reports based on other murine models of inflammation [22], we found large numbers of iNOS-expressing Gr-1^+^ myeloid cells within LYVE-1^+^ lymphatic vessels in the PLNs (Additional file 3). However, because it is unlikely that NO from these cells could affect LSMC contractions in the absence of an LEC barrier defect, we tested the hypothesis that LECs are a primary source of iNOS. We found that in vitro purified PDPN^+^ LECs from TNF-Tg mouse synovium have a significant 8-fold higher *iNOS* mRNA expression level than WT LECs (Fig. 1a and b) and that TNF (≥0.1 ng/ml) stimulation of WT LECs significantly increased *iNOS* levels by 40-fold (Fig. 1c). IHC confirmed that PDPN^+^ LECs in efferent lymphatic vessels from arthritic ankles from TNF-Tg mice express iNOS (Fig. 1d).

**Fig. 2:**
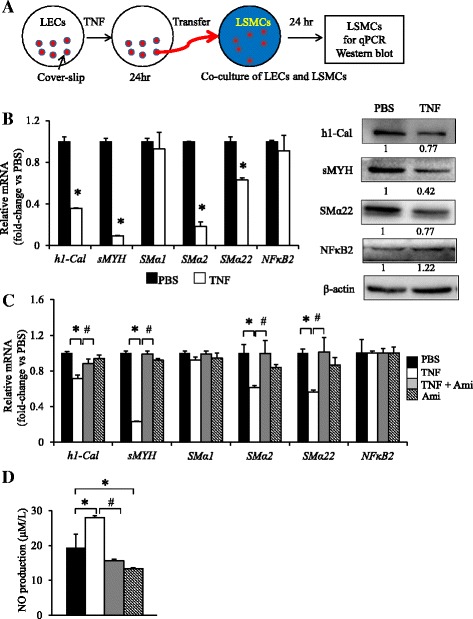
Nitric oxide (NO) from tumor necrosis factor (TNF)-treated lymphatic endothelial cells (LECs) reduces the expression levels of functional muscle genes in lymphatic smooth muscle cells (LSMCs), which is prevented by a selective inducible nitric oxide synthase (iNOS) inhibitor. **a** A schematic cartoon of the coculture model used to assess paracrine effects of NO induced by TNF in LECs on LSMC gene expression. **b** The effect of 1 ng/ml TNF on expression levels of functional muscle genes in LSMCs was determined by quantitative polymerase chain reaction (qPCR) and Western blot analysis. The signal intensity of bands on the Western blot was quantified by densitometry. **c** LECs were treated with TNF as in (**a**) with or without the selective NOS inhibitor aminoguanidine hemisulfate salt (Ami) or Ami alone and then cocultured with LSMCs for another 24 h. The expression levels of functional muscle genes were assessed by qPCR. The fold changes were calculated using phosphate-buffered saline (PBS)-treated sample values as 1. Values are mean ± standard deviation (SD) of three samples. *p < 0.05 vs. PBS-treated samples. **d** NO levels in the conditioned medium of TNF-treated LECs with or without different amounts of Ami or Ami alone were measured by using a Griess method. Values are mean ± SD of three samples. The experiments were repeated twice with similar results. *p < 0.05 vs. samples without Ami and TNF-treated; ^#^ *p* < 0.05 vs. TNF-treated samples. *NF-κB2* nuclear factor κB2, *sMYH* smooth muscle myosin heavy chain, *SMα* smooth muscle actin

To determine if TNF-Tg LECs affect LSMC function in vitro, we established a coculture system by treating LECs with TNF, coculturing the stimulated LECs with LSMCs, and then examining muscle-related gene expression in the LSMCs (Fig. 2a). We found that paracrine factors from the TNF-stimulated LECs significantly reduced expression of multiple functional muscle genes, including h1-Cal, smooth muscle myosin heavy chain 11, SMα2, and SMα22 (Fig. 2b). These inhibitory effects appeared to be NO-dependent because addition of the iNOS inhibitor Ami to the cocultures fully restored LSMC gene expression (Fig. 2c) and returned NO levels to baseline (Fig. 2d). Interestingly, TNF stimulation did not affect SMα1 mRNA level, which we subsequently used as an LSMC-specific gene control together with a non–cell-specific NF-κB2 control.

### Selective iNOS inhibition partially restores lymphatic vessel contractions and lymph drainage in TNF-Tg mice with severe arthritis

**Fig. 3:**
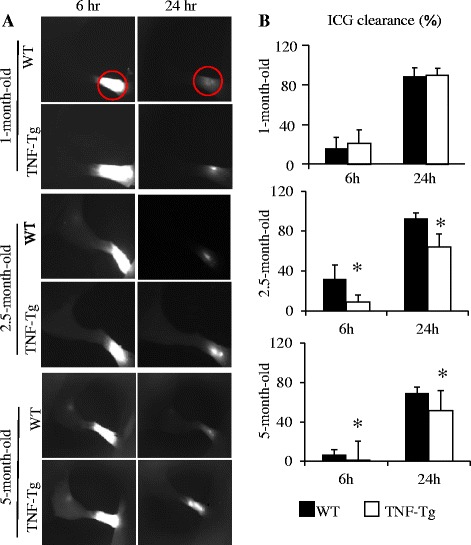
Decreased lymphatic clearance in tumor necrosis factor–transgenic (TNF-Tg) mice is associated with the progression of arthritis. Indocyanine green ([ICG] 0.1 mg/ml in 6 μl) was injected intradermally into the footpads of 1-, 2.5-, and 5-month-old TNF-Tg mice and their wild-type (WT) littermates. Lymphatic vessels of the entire leg were examined using near-infrared ICG imaging immediately and at 6 and 24 h post-ICG administration. The ICG fluorescence signal intensity at the footpads (outlined by the *red circles*) was recorded. The ICG clearance was calculated. Shown are a representative ICG signal (**a**) and the percentage of ICG clearance (**b**). Values are mean ± standard deviation of four or five mice. *p < 0.05 vs. WT mice

**Fig. 4:**
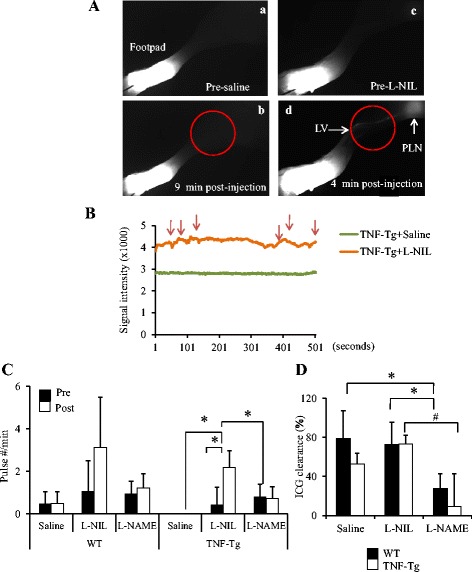
Differential effects of selective inducible nitric oxide synthase (iNOS) inhibition vs. total nitric oxide synthase (NOS) inhibition on lymphatic vessel contractions and lymph drainage in tumor necrosis factor–transgenic (TNF-Tg) mice with flaring arthritis. TNF-Tg mice more than 5 months old with severe ankle arthritis were used. **a** TNF-Tg mice (*n* = 5) were subjected to real-time near-infrared indocyanine green (NIR-ICG) imaging to quantify the afferent lymphatic pulse before, during, and following a direct injection of saline (**a**, **b**) or L-N^6^-(1-iminoethyl)lysine 5-tetrazole-amide (L-NIL) (**c**, **d**) into the footpad. Representative NIR-ICG images illustrate the lack of ICG lymphatic drainage from the injection site in the footpad to the region of the popliteal lymph node at 1 h postinjection (**a**, **c**). No remarkable changes were observed 9 minutes after saline injection (**b**). However, ICG was detected in the primary lymphatic vessel (LV; *arrow*) and the popliteal lymph node (PLN; *arrowhead*) within 4 minutes after L-NIL injection (**d**). **b** The lymphatic vessel contraction frequency was determined by assessing pulses in the NIR-ICG signal intensity in the region of interest (*red circles*), which was graphed over time during the imaging session. Note the recovery of lymphatic pulses (*red arrows*) after the iNOS inhibitor L-NIL (*green line*) injection, while saline did not affect lymphatic contraction frequency (*orange line*). **c** TNF-Tg mice and their wild-type (WT) littermates (*n* = 4) underwent pre- and posttreatment NIR-ICG imaging to assess the effects of 6 weeks of saline, L-NIL, and *N*_ω_-nitro-L-arginine methyl ester (l-NAME) on lymphatic vessel contractions (**c**) and percentage ICG clearance (**d**). Figure 4c, *P<0.05 between groups.  Figure 4d, *P<0.05 vs WT. #P<0.05 vs. TNF-Tg

We have shown previously that TNF-Tg mice with severe arthritis typically have reduced or loss of lymphatic vessel contractions and decreased lymphatic flow from inflamed joints, but the mechanisms involved are unknown [7, 8, 13, 37]. To elucidate these mechanisms, we first determined the initial onset of lymphatic dysfunction in the hind limbs of TNF-Tg mice using longitudinal NIR-ICG imaging (Fig. 3). We found a significant delay in ICG clearance from the footpads of TNF-Tg mice beginning approximately 2.5 months of age, as compared with their WT littermates. To assess the role of iNOS in these changes in the lower limbs of TNF-Tg mice with ankle arthritis, we examined the acute effects of local administration of the selective iNOS inhibitor l-NIL (Fig. 4a and b) and found that a single injection of l-NIL increased lymphatic flow and partially restored lymphatic contractions within 400 seconds of administration, while injection of saline had no effects.

To confirm that lymphatic function recovery was due to iNOS inhibition and to assess drug effects on normal lymphatic vessel function, we treated cohorts of TNF-Tg mice with severe ankle arthritis and their WT littermates with saline, L-NIL, and the nonspecific NOS inhibitor L-NAME for 6 weeks (Fig. 4c and Additional file 4). Unfortunately, these drug treatment regimens proved to be too toxic for assessment of lymphatic function, with a >50 % fatality rate in the L-NAME group and a >30 % fatality rate in the l-NIL group beyond 6 weeks of treatment. However, NIR-ICG imaging at 6 weeks posttreatment demonstrated that L-NIL significantly increased the lymphatic pulse in TNF-Tg mice (from 0.42 ± 0.84 to 2.19 ± 0.77 pulses/minute; *p* < 0.05), but not in their WT littermates (0.96 ± 1.42 to 3.13 ± 2.36 pulses/minute; *p* = 0.08). In contrast, saline or L-NAME did not change the lymphatic pulse in either TNF-Tg or WT mice. Similarly, L-NIL significantly increased lymph drainage in TNF-Tg mice compared with saline (73.5 ± 8.9 % vs. 53.2 ± 12.7 % ICG clearance; *p* < 0.05), while L-NAME decreased lymphatic drainage in both WT and TNF-Tg mice. These data indicate that only selective iNOS inhibition by L-NIL significantly increased lymphatic vessel contractions and lymphatic flow in TNF-Tg mice, but not in WT littermates. The nonspecific NOS inhibition by L-NAME significantly decreased ICG clearance in both WT and TNF-Tg mice, consistent with a critical role for endothelial nitric oxide synthase (eNOS) signaling in basal lymphatic function.

### Ferulic acid therapy improves TNF-induced inflammatory erosive arthritis, which is associated with restored lymphatic vessel contractions, lymphatic flow, and LSMC gene expression

Given the toxicity challenges associated with selective iNOS inhibitors and our interest in understanding the mechanism of action of traditional Chinese medicines (TCMs) that have been used successfully to treat patients with RA who do not respond to disease-modifying antirheumatic drugs [42–46], we performed a drug screen in zebrafish to identify TCM-derived small molecules based on their ability to stimulate lymphatic vessel growth (data not shown). We found ferulic acid, an *Angelica sinensis* extract [47] used to treat patients with vascular disease and arthritis [33, 34, 48, 49], to be our lead candidate.

**Fig. 5:**
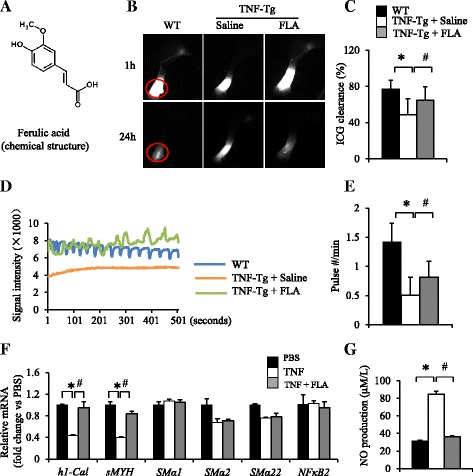
Ferulic acid (FLA) rescues impaired lymphatic function in tumor necrosis factor–transgenic (TNF-Tg) mice. Three-month-old TNF-Tg mice (*n* = 8/group, comprising 4 females and 4 males) were treated with FLA (20 mg/kg by gavage daily for 12 weeks) or saline and were subjected to near-infrared indocyanine green imaging (NIR-ICG) imaging. **a** Chemical structure of FLA. **b** Representative ICG images show that FLA increased ICG removal from the ankle area (*red circles*) 24 h after ICG injection. **c** Quantitation of percentage of ICG clearance. Values are mean ± standard deviation (SD) of 16–20 affected legs. **d** Lymphatic pulses were measured at the region of interest, as shown in Fig. 4. Histogram shows that FLA restored lymphatic pulses in TNF-Tg mice. **e** Quantitation of lymphatic pulses/minute. Values are mean ± SD of 9–11 affected legs from 5–7 mice. *p < 0.05 vs. wild-type (WT) mice, ^#^ *p* < 0.05 vs. TNF-Tg mice. **f** Lymphatic endothelial cells (LECs) were treated with TNF (as shown in Fig. 2c) with or without FLA for 24 h and then cocultured with lymphatic smooth muscle cells (LSMCs) for another 24 h. The expression levels of functional muscle genes were determined by quantitative polymerase chain reaction. The fold changes were calculated using the saline-treated sample value as 1. Values are mean ± SD of three samples. **g** Nitric oxide (NO) levels in the conditioned medium of TNF-treated LECs with or without FLA were measured by a Griess method as in Fig. 2. Values are mean ± SD of three samples. *p < 0.05 vs. saline-treated group, ^#^ *p* < 0.05 vs. TNF-treated group. *h1-Cal* h1-calponin, *mRNA* messenger RNA, *NF-κB2* nuclear factor κB2, *SMα* smooth muscle actin, *sMYH* smooth muscle myosin heavy chain

**Fig. 6:**
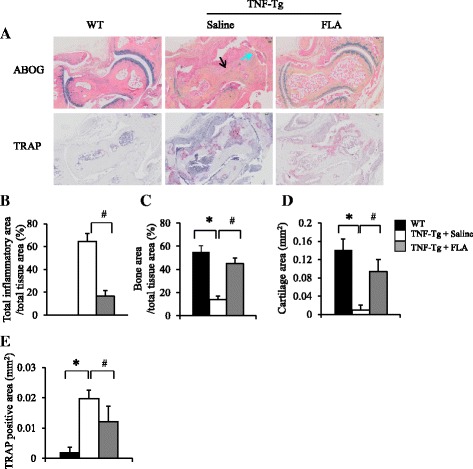
Ferulic acid (FLA) reduces joint tissue damage in tumor necrosis factor–transgenic (TNF-Tg) mice. Three-month-old TNF-Tg and wild-type (WT) mice were treated with FLA or saline, as described in Fig. 5. **a** Representative Alcian Blue/Orange G (ABOG)- and tartrate-resistant acid phosphatase (TRAP)-stained sections show decreased joint tissue damage, including decreased cartilage (*blue arrow*) and bone erosion (*black arrow*) and TRAP^+^ osteoclasts in an FLA-treated mouse which has relatively normal joint morphology. Quantitation of inflammatory area (**b**), bone area (**c**), cartilage area (**d**), and TRAP^+^ area (**e**). Values are the mean ± standard deviation of six to eight legs per group. *p < 0.05 vs. WT mice; ^#^ *p* < 0.05 vs. saline-treated TNF-Tg mice

To assess the effects of ferulic acid therapy on TNF-Tg mice with arthritis, we treated a cohort of TNF-Tg mice for 12 weeks and examined changes in lymphatic flow, lymphatic contraction frequency, LSMC gene expression, synovitis, and focal erosions by NIR-ICG imaging, qPCR, and histology. NIR-ICG imaging demonstrated that placebo-treated TNF-Tg mice had a significant 30 % decrease in lymph flow from their lower limbs, as well as a significant threefold decrease in lymphatic vessel contraction frequency, compared with their WT littermates (*p* < 0.05). In contrast, ferulic acid therapy restored lymph flow and lymphatic vessel contraction frequency in TNF-Tg mice to WT levels (Fig. 5a–e). In vitro coculture studies demonstrated that ferulic acid increased expression levels of the muscle functional genes *h1-calponinl* and *sMYH* in LSMCs (Fig. 5f) and completely inhibited TNF-induced NO production by LECs (Fig. 5g). Ferulic acid did not restore *SMα2* transcript levels in LSMCs, suggesting that the LEC paracrine factor that downregulates this gene is not NO. Histologic analyses of ankle tissues from these mice demonstrated that ferulic acid markedly reduced inflammatory erosive arthritis progression, as determined by significant decreases in inflammatory tissue area and osteoclast numbers, as well as significant increases in bone and cartilage area, compared with placebo controls (Fig. 6). Thus, ferulic acid amelioration of arthritis is associated with normalization of draining lymphatic function.

## Discussion

Although RA is still broadly considered to be an autoimmune disease, largely based on diagnostic autoantibodies, a more contemporary view is that RA is a complex disease syndrome in which variable host and environmental factors activate several pathogenic pathways that result in a similar clinical phenotype [50]. One prominent clinical phenotype of RA is sudden-onset painful synovitis in affected joints, even in patients responsive to anti-TNF treatment. This is commonly referred to as *arthritic flare*, which enigmatically waxes and wanes over decades of systemic disease without detectable changes in autoimmunity. One mechanism we have proposed to account for the development of arthritic flare involves loss of lymph egress from diseased joints [7–9]. Consistent with this, we reported lymphatic draining dysfunction in several animal models of inflammatory erosive arthritis and showed that lymphatic drainage is negatively correlated with the severity of joint tissue damage [8, 13, 37, 41, 51]. Furthermore, we demonstrate in the present study that the progression of ankle arthritis occurs coincident with the loss of lymphatic function and lymph flow from the adjacent tissue. Thus, there is now a large body of experimental evidence to support the theory that arthritis is associated with lymphatic dysfunction. However, the precise cellular and molecular mechanisms underlying this lymphatic dysfunction remain to be elucidated.

It was reported recently that the NO–NOS axis directly decreases the contractile capacity of LSMCs [22, 23] and thereby reduces the drainage function of lymphatic vessels. These findings are based on mouse models of acute inflammation in which the inflammatory responses typically resolve spontaneously within 1 week and macrophages are the major source of iNOS. In contrast to these models, the inflammation in joints of TNF-Tg mice is long-standing and progressive, beginning at 2 months after birth, and worsens without resolving spontaneously as the mice age. Thus, TNF-Tg mice have features similar to those seen in patients with RA with uncontrolled active disease.

We initially sought to identify the iNOS-expressing cells in efferent lymphatic vessels draining affected joints in TNF-Tg mice that could be responsible for the loss of lymphatic contractions. We observed vessels draining inflamed paws. Consistent with published reports, we found high levels of iNOS expressing Gr-1^+^ myeloid cells within the lymphatic vessels. However, because NO has an extremely short half-life and the numbers of these intraluminal myeloid cells are small, we think that they are an unlikely source of the NO that inhibits LSMCs in the absence of a major lymphatic endothelial barrier defect. Researchers in previous studies reported that eNOS from endothelial cells induces low levels of NO production to maintain vascular contractile activity [20, 21]. Thus, we compared eNOS and iNOS expression in LECs in synovial tissues around TNF-Tg mouse joints and found much higher levels of iNOS than eNOS (Fig. 1), suggesting that iNOS may play a more important role than eNOS in NO production under chronic inflammatory conditions.

Another interesting finding is that very low doses of TNF (0.1 ng/ml) induced a high level of expression of iNOS in LECs, indicating that LECs are very sensitive to iNOS induced locally by inflammatory cytokines. We reported previously that the circulating human TNF levels in TNF-Tg mice are about 0.1–0.2 ng/ml [36, 52]. Thus, the dose at which TNF could increase iNOS expression in LECs is comparable to in vivo TNF levels in TNF-Tg mice. Our novel findings that TNF directly stimulates iNOS and NO production in LECs and that this iNOS-induced NO decreases LSMC gene expression and lymphatic function in vivo provide a plausible biological mechanism for the loss of lymphatic vessel contractions and decreased lymph flow observed during the progression of arthritis.

LECs may be exposed to TNF in vivo by a number of mechanisms. For example, TNF levels will be increased in the interstitial fluid draining from inflamed joints when it passes into collecting lymphatic vessels as well as in soft tissues generally because of high serum TNF levels in these mice [14]. Another source of TNF is likely from macrophages that pass or get trapped in the lymphatic vessels, as we have described recently [35, 36]. Because in inflamed joints iNOS can be produced by other cell types, such as macrophages and synovial cells, it is necessary to use mice with iNOS knocked out specifically in these and other cell types to fully determine the role of iNOS produced by LECs in lymphatic dysfunction in RA. TNF and other proinflammatory cytokines could also inhibit LSMCs directly. Thus, multiple factors likely contribute to the reduced lymphatic drainage in RA.

Our study clearly indicates the contribution of NO from LECs to inhibition of LSMC function in the setting of TNF overexpression, which provides experimental evidence for the use of NOS inhibitors in the treatment of RA. It is important to note that iNOS is a well-established target for RA therapy, as the efficacy of two selective inhibitors—l-NIL [53] and GW274150 [54]—has already been demonstrated to prevent collagen-induced arthritis. Moreover, GW274150 has recently been evaluated in a phase IIa clinical trial for early RA, in which the drug showed a trend toward reduction in synovial thickness (33 %; *p* = 0.072) and synovial vascularity (42 %; *p* = 0.075) vs. placebo [55]. However, we found that TNF-Tg mice could not tolerate the published dosing regimens of l-NAME and l-NIL. Because the primary cause of early death (6–12 months of age) of TNF-Tg mice is pulmonary hypertension, which is also the primary toxicity associated with NO inhibitors [56], we speculate that the combined TNF-induced pathology and adverse drug effects lead to unacceptable side effects that preclude study of potent NOS inhibitors in this model. Thus, we evaluated other known drugs that have less toxicity. In this study, we chose to investigate TCM derivatives, which have an established highly favorable safety and tolerability profile in humans. Following identification of ferulic acid as our lead candidate in a zebrafish screen, we demonstrated that treatment of TNF-Tg mice with this drug significantly (1) increased lymphatic contraction and transport, (2) blocked TNF-induced LEC NO production and LSMC inhibition, and (3) reduced joint inflammation and bone and cartilage erosion. Given this initial proof of concept and that ferulic acid is used to treat patients with vascular diseases and chronic inflammation [33, 34, 48, 49], further studies are warranted to evaluate this potentially highly cost-effective therapy for RA. Another approach to reducing toxicity is to generate targeted agents that could be delivered to joints locally, resulting in enhanced local concentrations and reduced systemic toxicity, such as nanoparticle-embedded drugs, which is currently under investigation in our laboratory.

## Conclusions

We have demonstrated that dysfunction of efferent lymphatic vessels from inflamed hind limb joints in TNF-Tg mice involves TNF-induced iNOS expression and NO production by LECs, which affects LSMCs and results in decreased lymphatic vessel contractions and lymph drainage. iNOS inhibitors, and especially ferulic acid therapy, ameliorate TNF-induced arthritis, which is associated with restoration of lymphatic vessel contractions and drainage. Thus, inhibition of NO production and improvement of lymphatic vessel draining function together represent a potential therapeutic strategy for chronic inflammatory arthritis. It also represents a new mechanism of action for some established drugs that have been used to treat patients with inflammatory arthritis.

## Additional files

Additional file 1: Figure S1. HPLC assessment of the purity of the experimental ferulic acid. The purity of the ferulic acid used in the experiments used in this study (*red line*) was determined to be >98 % homogeneous by HPLC versus a known standard (*blue line*). (PPTX 45 kb) Additional file 2: Table S1. Sequences of primers used in real-time polymerase chain reactions. (PPTX 93 kb) Additional file 3: Figure S2. Large numbers of iNOS-expressing Gr-1^+^ monocytes in PLN and efferent lymphatic vessels from arthritic ankles in TNF-Tg mice. PLNs were harvested from 5-month-old TNF-Tg mice with frank ankle arthritis and from their nonarthritic WT littermates (*n* = 5), and the tissues were processed for fresh frozen IHC using PE-conjugated Gr-1, FITC-conjugated LYVE-1, and FITC-conjugated iNOS antibodies. DAPI was used to counterstain nuclei (*blue*) before fluorescence microscopy. Representative images (original magnification × 5) of WT (**a**) and TNF-Tg (**b**) PLN sections illustrate the typical increase in size of TNF-Tg PLNs due to increased cellularity and lymphangiogenesis. **c** A representative immunofluorescence image (original magnification × 20) of the boxed region in (**b**) is shown to illustrate the prevalence of Gr-1 and iNOS double-positive monocytes within LYVE-1^+^ lymphatic vessels in TNF-Tg PLN, which were not present in PLN from nonarthritic WT mice. (PPTX 893 kb) Additional file 4: Figure S3. Effects of selective (l-NIL) and nonselective (l-NAME) NOS inhibitors on the lymphatic pulse with WT and TNF-Tg mice. The number of lymphatic vessel contractions (Pulse #) per minute of ICG filled lymphatic vessel afferent to the PLN of WT and TNF-Tg mice, pre- and posttreated with saline, l-NIL, or l-NAME, was quantified from NIR movies as described in the Methods section. Each data point represents the mean for an individual mouse in the group with the SEM of the group. (PPTX 61 kb)

## Abbreviations

ABOG: Alcian Blue/Orange G
Ami: aminoguanidine hemisulfate salt
BSA: bovine serum albumin
C_t_: threshold cycle
DAPI: 4′,6-diamidino-2-phenylindole
EDTA: ethylenediaminetetraacetic acid
eNOS: endothelial nitric oxide synthase
FBS: fetal bovine serum
FITC: fluorescein isothiocyanate
FLA: ferulic acid
h1-Cal: h1-calponin
H&E: hematoxylin and eosin
ICG: indocyanine green
IHC: immunohistochemistry
iNOS: inducible nitric oxide synthase
LEC: lymphatic endothelial cell
LED: light-emitting diode
l-NAME: *N*_ω_-nitro-l-arginine methyl ester
l-NIL: l-N^6^-(1-iminoethyl)lysine 5-tetrazole-amide
LSMC: lymphatic smooth muscle cell
LV: lymphatic vessel
LYVE-1: lymphatic vessel endothelial hyaluronan receptor 1
mRNA: messenger RNA
NF-κB2: nuclear factor κB2
NIR: near-infrared
NO: nitric oxide
PBS: phosphate-buffered saline
PCR: polymerase chain reaction
PDPN: podoplanin
PE: phycoerythrin
PLN: popliteal lymph node
RA: rheumatoid arthritis
ROI: region of interest
RT: room temperature
SD: standard deviation
SMα: smooth muscle actin
sMYH: smooth muscle myosin heavy chain
TCM: traditional Chinese medicine
TNF-Tg: tumor necrosis factor–transgenic
TRAP: tartrate-resistant acid phosphatase
VEGFR3: vascular endothelial growth factor receptor 3
WT: wild type

## References

[1] Zawieja D (2005). Lymphatic biology and the microcirculation: past, present and future. Microcirculation.

[2] Wilting J, Becker J, Buttler K, Weich HA (2009). Lymphatics and inflammation. Curr Med Chem.

[3] Olszewski WL, Pazdur J, Kubasiewicz E, Zaleska M, Cooke CJ, Miller NE (2001). Lymph draining from foot joints in rheumatoid arthritis provides insight into local cytokine and chemokine production and transport to lymph nodes. Arthritis Rheum.

[4] Wauke K, Nagashima M, Ishiwata T, Asano G, Yoshino S (2002). Expression and localization of vascular endothelial growth factor-C in rheumatoid arthritis synovial tissue. J Rheumatol.

[5] Zhang Q, Lu Y, Proulx ST, Guo R, Yao Z, Schwarz EM (2007). Increased lymphangiogenesis in joints of mice with inflammatory arthritis. Arthritis Res Ther.

[6] Polzer K, Baeten D, Soleiman A, Distler J, Gerlag DM, Tak PP (2008). Tumour necrosis factor blockade increases lymphangiogenesis in murine and human arthritic joints. Ann Rheum Dis.

[7] Proulx ST, Kwok E, You Z, Beck CA, Shealy DJ, Ritchlin CT (2007). MRI and quantification of draining lymph node function in inflammatory arthritis. Ann N Y Acad Sci..

[8] Li J, Kuzin I, Moshkani S, Proulx ST, Xing L, Skrombolas D (2010). Expanded CD23^+^/CD21^hi^ B cells in inflamed lymph nodes are associated with the onset of inflammatory-erosive arthritis in TNF-transgenic mice and are targets of anti-CD20 therapy. J Immunol.

[9] Benaglio F, Vitolo B, Scarabelli M, Binda E, Bugatti S, Caporali R (2015). The draining lymph node in rheumatoid arthritis: current concepts and research perspectives. Biomed Res Int..

[10] Keffer J, Probert L, Cazlaris H, Georgopoulos S, Kaslaris E, Kioussis D (1991). Transgenic mice expressing human tumour necrosis factor: a predictive genetic model of arthritis. EMBO J.

[11] Kouskoff V, Korganow AS, Duchatelle V, Degott C, Benoist C, Mathis D (1996). Organ-specific disease provoked by systemic autoimmunity. Cell.

[12] Bouta EM, Li J, Ju Y, Brown EB, Ritchlin CT, Xing L (2015). The role of the lymphatic system in inflammatory-erosive arthritis. Semin Cell Dev Biol..

[13] Zhou Q, Wood R, Schwarz EM, Wang YJ, Xing L (2010). Near-infrared lymphatic imaging demonstrates the dynamics of lymph flow and lymphangiogenesis during the acute versus chronic phases of arthritis in mice. Arthritis Rheum.

[14] Li J, Ju Y, Bouta EM, Xing L, Wood RW, Kuzin I (2013). Efficacy of B cell depletion therapy for murine joint arthritis flare is associated with increased lymphatic flow. Arthritis Rheum.

[15] Bouta EM, Wood RW, Perry SW, Brown EB, Ritchlin CT, Xing L (2011). Measuring intranodal pressure and lymph viscosity to elucidate mechanisms of arthritic flare and therapeutic outcomes. Ann N Y Acad Sci..

[16] van der Flier A, Badu-Nkansah K, Whittaker CA, Crowley D, Bronson RT, Lacy-Hulbert A (2010). Endothelial α5 and αv integrins cooperate in remodeling of the vasculature during development. Development.

[17] Zhou F, Chang Z, Zhang L, Hong YK, Shen B, Wang B (2010). Akt/protein kinase B is required for lymphatic network formation, remodeling, and valve development. Am J Pathol.

[18] von der Weid PY, Zawieja DC (2004). Lymphatic smooth muscle: the motor unit of lymph drainage. Int J Biochem Cell Biol.

[19] Van Helden DF (1993). Pacemaker potentials in lymphatic smooth muscle of the guinea-pig mesentery. J Physiol..

[20] Mathias R, von der Weid PY (2013). Involvement of the NO-cGMP-K_ATP_ channel pathway in the mesenteric lymphatic pump dysfunction observed in the guinea pig model of TNBS-induced ileitis. Am J Physiol Gastrointest Liver Physiol.

[21] Schmid-Schonbein GW (2012). Nitric oxide (NO) side of lymphatic flow and immune surveillance. Proc Natl Acad Sci U S A.

[22] Liao S, Cheng G, Conner DA, Huang Y, Kucherlapati RS, Munn LL (2011). Impaired lymphatic contraction associated with immunosuppression. Proc Natl Acad Sci U S A.

[23] Scallan JP, Davis MJ (2013). Genetic removal of basal nitric oxide enhances contractile activity in isolated murine collecting lymphatic vessels. J Physiol.

[24] Farrell AJ, Blake DR, Palmer RM, Moncada S (1992). Increased concentrations of nitrite in synovial fluid and serum samples suggest increased nitric oxide synthesis in rheumatic diseases. Ann Rheum Dis.

[25] Grabowski PS, England AJ, Dykhuizen R, Copland M, Benjamin N, Reid DM (1996). Elevated nitric oxide production in rheumatoid arthritis: detection using the fasting urinary nitrate:creatinine ratio. Arthritis Rheum.

[26] McInnes IB, Leung BP, Field M, Wei XQ, Huang FP, Sturrock RD (1996). Production of nitric oxide in the synovial membrane of rheumatoid and osteoarthritis patients. J Exp Med.

[27] Sakurai H, Kohsaka H, Liu MF, Higashiyama H, Hirata Y, Kanno K (1995). Nitric oxide production and inducible nitric oxide synthase expression in inflammatory arthritides. J Clin Invest.

[28] Connor JR, Manning PT, Settle SL, Moore WM, Jerome GM, Webber RK (1995). Suppression of adjuvant-induced arthritis by selective inhibition of inducible nitric oxide synthase. Eur J Pharmacol.

[29] Grabowski PS, Wright PK, Van ’t Hof RJ, Helfrich MH, Ohshima H, Ralston SH (1997). Immunolocalization of inducible nitric oxide synthase in synovium and cartilage in rheumatoid arthritis and osteoarthritis. Br J Rheumatol.

[30] Leak LV, Cadet JL, Griffin CP, Richardson K (1995). Nitric oxide production by lymphatic endothelial cells in vitro. Biochem Biophys Res Commun.

[31] McCartney-Francis N, Allen JB, Mizel DE, Albina JE, Xie QW, Nathan CF (1993). Suppression of arthritis by an inhibitor of nitric oxide synthase. J Exp Med.

[32] Ribera J, Pauta M, Melgar-Lesmes P, Tugues S, Fernández-Varo G, Held KF (2013). Increased nitric oxide production in lymphatic endothelial cells causes impairment of lymphatic drainage in cirrhotic rats. Gut.

[33] Yang CL, Or TC, Ho MH, Lau AS (2013). Scientific basis of botanical medicine as alternative remedies for rheumatoid arthritis. Clin Rev Allergy Immunol.

[34] Lee WY, Chen HY, Chen KC, Chen CY (2014). Treatment of rheumatoid arthritis with traditional Chinese medicine. Biomed Res Int..

[35] Guo R, Zhou Q, Proulx ST, Wood R, Ji RC, Ritchlin CT (2009). Inhibition of lymphangiogenesis and lymphatic drainage via vascular endothelial growth factor receptor 3 blockade increases the severity of inflammation in a mouse model of chronic inflammatory arthritis. Arthritis Rheum.

[36] Li P, Schwarz EM, O’Keefe RJ, Ma L, Looney RJ, Ritchlin CT (2004). Systemic tumor necrosis factor α mediates an increase in peripheral CD11b^high^ osteoclast precursors in tumor necrosis factor α-transgenic mice. Arthritis Rheum.

[37] Proulx ST, Kwok E, Beck CA, Shealy DJ, Ritchlin CT, Award H, et al. Longitudinal assessment of synovial, lymph node, and bone volumes in inflammatory arthritis in mice using in vivo MRI and micro-CT. Arthritis Rheum. 2007;56(12):4024-37. 10.1002/art.23128 PMC2662386 18050199

[38] Yu Y, Fan SM, Yuan SJ, Tashiro S, Onodera S, Ikejima T (2012). Nitric oxide (•NO) generation but not ROS plays a major role in silibinin-induced autophagic and apoptotic death in human epidermoid carcinoma A431 cells. Free Radic Res.

[39] Muthuchamy M, Gashev A, Boswell N, Dawson N, Zawieja D (2003). Molecular and functional analyses of the contractile apparatus in lymphatic muscle. FASEB J.

[40] Sironi M, Conti A, Bernasconi S, Fra AM, Pasqualini F, Nebuloni M (2006). Generation and characterization of a mouse lymphatic endothelial cell line. Cell Tissue Res.

[41] Li J, Zhou Q, Wood R, Kuzin I, Bottaro A, Ritchlin C (2011). CD23^+^/CD21^hi^ B cell translocation and ipsilateral lymph node collapse is associated with asymmetric arthritic flare in TNF-Tg mice. Arthritis Res Ther.

[42] Zhang P, Li J, Han Y, Yu XW, Qin L (2010). Traditional Chinese medicine in the treatment of rheumatoid arthritis: a general review. Rheumatol Int.

[43] Zhao XX, Peng C, Zhang H, Qin LP (2012). *Sinomenium acutum*: a review of chemistry, pharmacology, pharmacokinetics, and clinical use. Pharm Biol.

[44] Ramgolam V, Ang SG, Lai YH, Loh CS, Yap HK (2000). Traditional Chinese medicines as immunosuppressive agents. Ann Acad Med Singapore.

[45] Ho LJ, Lai JH (2004). Chinese herbs as immunomodulators and potential disease-modifying antirheumatic drugs in autoimmune disorders. Curr Drug Metab.

[46] Wang T, Wei Z, Dou Y, Yang Y, Leng D, Kong L (2015). Intestinal interleukin-10 mobilization as a contributor to the anti-arthritis effect of orally administered madecassoside: a unique action mode of saponin compounds with poor bioavailability. Biochem Pharmacol.

[47] Yao H, Shi P, Shao Q, Fan X (2011). Chemical fingerprinting and quantitative analysis of a *Panax notoginseng* preparation using HPLC-UV and HPLC-MS. Chin Med..

[48] Ye HZ, Zheng CS, Xu XJ, Wu MX, Liu XX (2011). Potential synergistic and multitarget effect of herbal pair *Chuanxiong Rhizome-Paeonia Albifora Pall* on osteoarthritis disease: a computational pharmacology approach. Chin J Integr Med.

[49] Jung SM, Schumacher HR, Kim H, Kim M, Lee SH, Pessler F (2007). Reduction of urate crystal-induced inflammation by root extracts from traditional oriental medicinal plants: elevation of prostaglandin D_2_ levels. Arthritis Res Ther.

[50] Firestein GS (2014). The disease formerly known as rheumatoid arthritis. Arthritis Res Ther.

[51] Zhou Q, Guo R, Wood R, Boyce BF, Liang Q, Wang YJ (2011). Vascular endothelial growth factor C attenuates joint damage in chronic inflammatory arthritis by accelerating local lymphatic drainage in mice. Arthritis Rheum.

[52] Yao Z, Xing L, Boyce BF (2009). NF-κB p100 limits TNF-induced bone resorption in mice by a TRAF3-dependent mechanism. J Clin Investig.

[53] Vermeire K, Thielemans L, Matthys P, Billiau A (2000). The effects of NO synthase inhibitors on murine collagen-induced arthritis do not support a role of NO in the protective effect of IFN-γ. J Leukoc Biol.

[54] Cuzzocrea S, Chatterjee PK, Mazzon E, McDonald MC, Dugo L, Di Paola R (2002). Beneficial effects of GW274150, a novel, potent and selective inhibitor of iNOS activity, in a rodent model of collagen-induced arthritis. Eur J Pharmacol.

[55] Seymour M, Petavy F, Chiesa F, Perry H, Lukey PT, Binks M (2012). Ultrasonographic measures of synovitis in an early phase clinical trial: a double-blind, randomised, placebo and comparator controlled phase IIa trial of GW274150 (a selective inducible nitric oxide synthase inhibitor) in rheumatoid arthritis. Clin Exp Rheumatol.

[56] Tisljar M, Grabarević Z, Artuković B, Dzaja P, Cenan S, Zelenika TA (2011). The impact of l-NAME and l-arginine chronic toxicity induced lesions on ascites—pulmonary hypertension syndrome development in broiler chickens. Coll Antropol.

