# Supplementary figures and images for: Lymphatic endothelial cells efferent to inflamed joints produce iNOS and inhibit lymphatic vessel contraction and drainage in TNF-induced arthritis in mice
Source: Arthritis Res Ther. 2016 Mar 12;18:62. doi: 10.1186/s13075-016-0963-8 (PMC4789262; doi:10.1186/s13075-016-0963-8)

## Slide 1
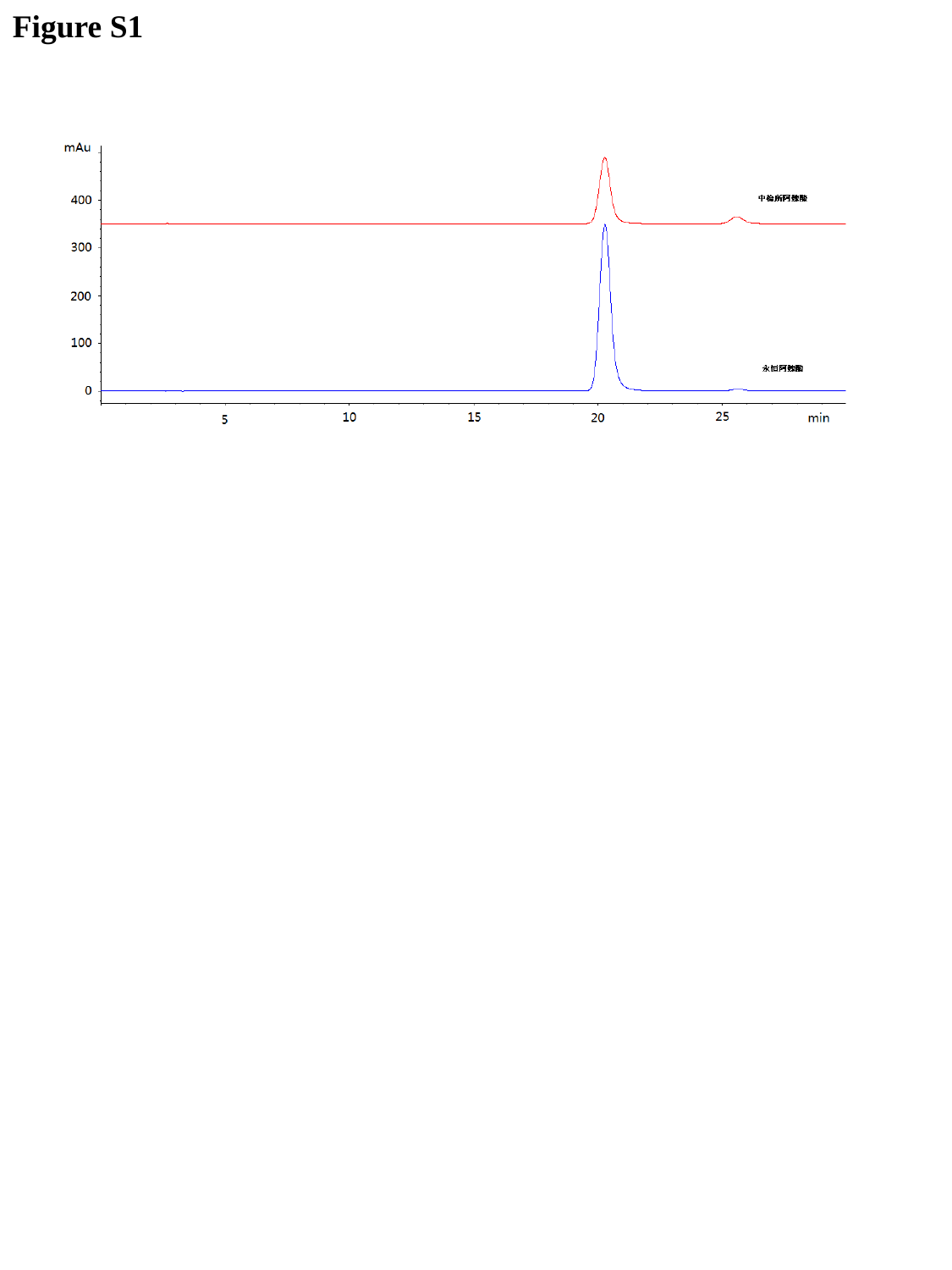

Figure S1

Supplement: Additional file 1: Figure S1. — HPLC assessment of the purity of the experimental ferulic acid. The purity of the ferulic acid used in the experiments used in this study (red line) was determined to be >98 % homogeneous by HPLC versus a known standard (blue line). (PPTX 45 kb) [file 13075_2016_963_MOESM1_ESM.pptx]

## Slide 1
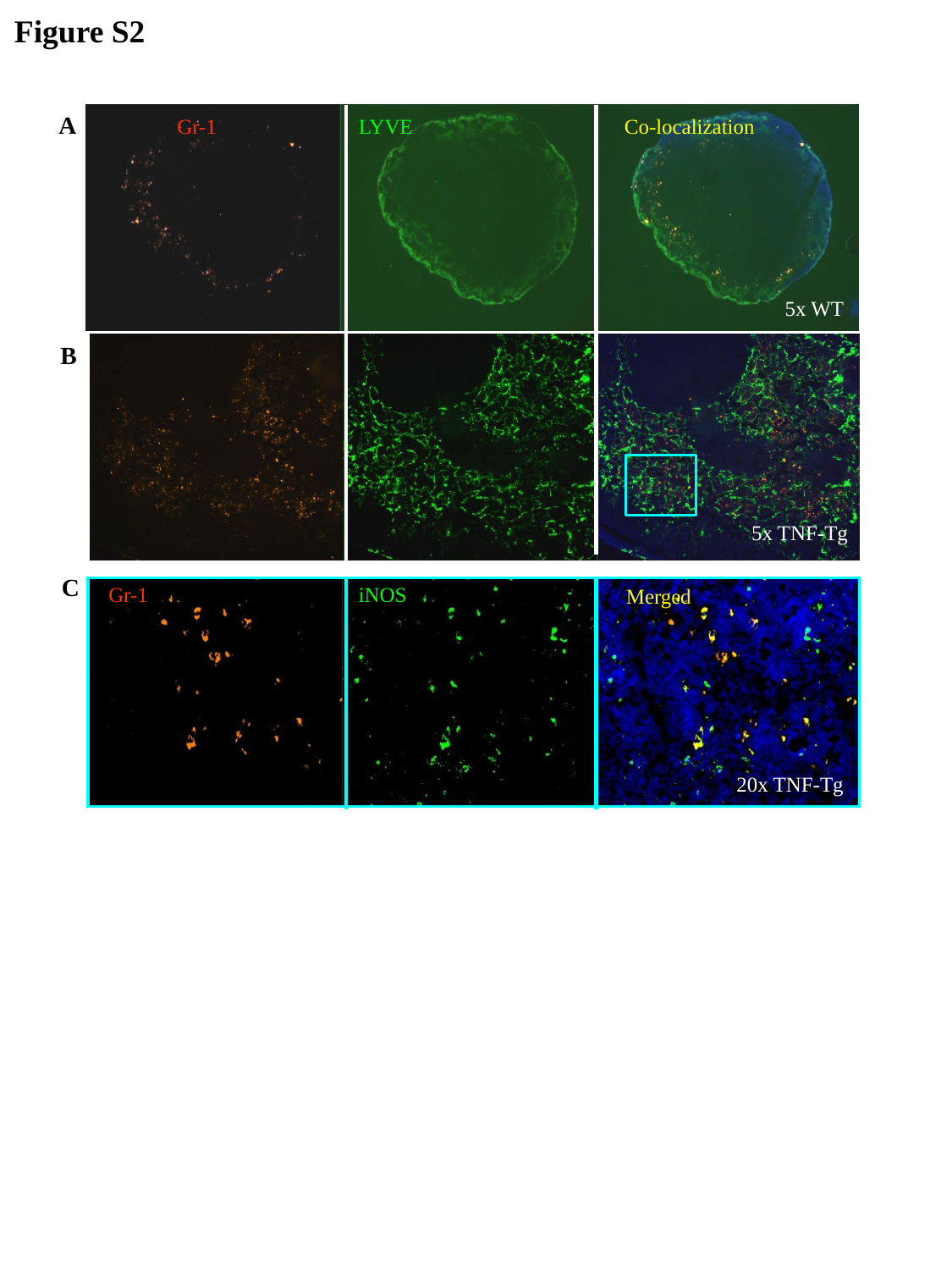

Figure S2
A
Gr-1
LYVE
Co-localization
5x WT
B
5x TNF-Tg
C
Gr-1
iNOS
Merged
20x TNF-Tg

Supplement: Additional file 3: Figure S2. — Large numbers of iNOS-expressing Gr-1+ monocytes in PLN and efferent lymphatic vessels from arthritic ankles in TNF-Tg mice. PLNs were harvested from 5-month-old TNF-Tg mice with frank ankle arthritis and from their nonarthritic WT littermates (n = 5), and the tissues were processed for fresh frozen IHC using PE-conjugated Gr-1, FITC-conjugated LYVE-1, and FITC-conjugated iNOS antibodies. DAPI was used to counterstain nuclei (blue) before fluorescence microscopy. Representative images (original magnification × 5) of WT (a) and TNF-Tg (b) PLN sections illustrate the typical increase in size of TNF-Tg PLNs due to increased cellularity and lymphangiogenesis. c A representative immunofluorescence image (original magnification × 20) of the boxed region in (b) is shown to illustrate the prevalence of Gr-1 and iNOS double-positive monocytes within LYVE-1+ lymphatic vessels in TNF-Tg PLN, which were not present in PLN from nonarthritic WT mice. (PPTX 893 kb) [file 13075_2016_963_MOESM3_ESM.pptx]

## Slide 1
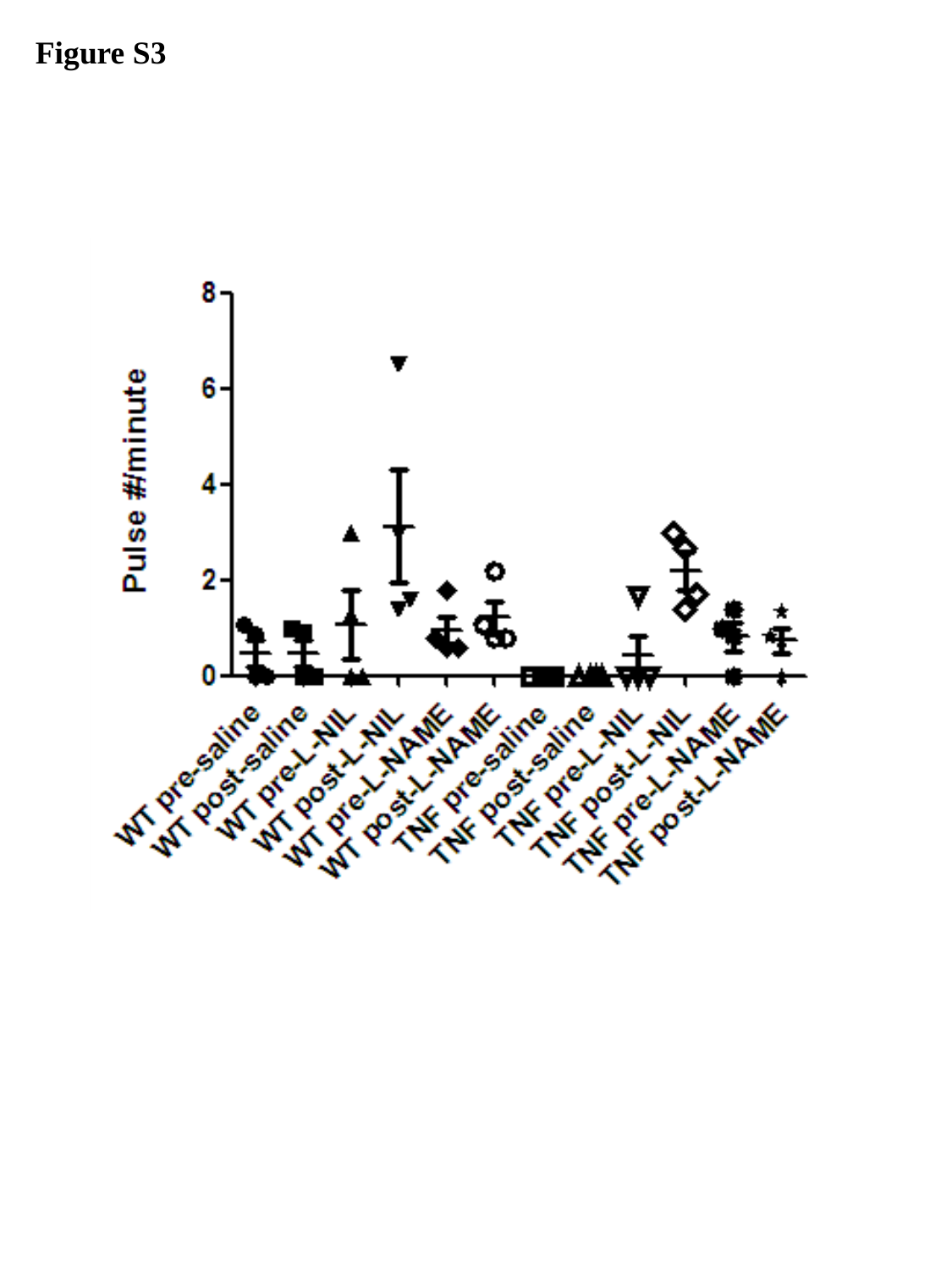

Figure S3

Supplement: Additional file 4: Figure S3. — Effects of selective (l-NIL) and nonselective (l-NAME) NOS inhibitors on the lymphatic pulse with WT and TNF-Tg mice. The number of lymphatic vessel contractions (Pulse #) per minute of ICG filled lymphatic vessel afferent to the PLN of WT and TNF-Tg mice, pre- and posttreated with saline, l-NIL, or l-NAME, was quantified from NIR movies as described in the Methods section. Each data point represents the mean for an individual mouse in the group with the SEM of the group. (PPTX 61 kb) [file 13075_2016_963_MOESM4_ESM.pptx]
