# Supplementary material for: Lymphatic endothelial cells efferent to inflamed joints produce iNOS and inhibit lymphatic vessel contraction and drainage in TNF-induced arthritis in mice
Source: Arthritis Res Ther. 2016 Mar 12;18:62. doi: 10.1186/s13075-016-0963-8 (PMC4789262; doi:10.1186/s13075-016-0963-8)
Supplement: Additional file 2: Table S1. — Sequences of primers used in real-time polymerase chain reactions. (PPTX 93 kb) [file 13075_2016_963_MOESM2_ESM.pptx]

## Slide 1
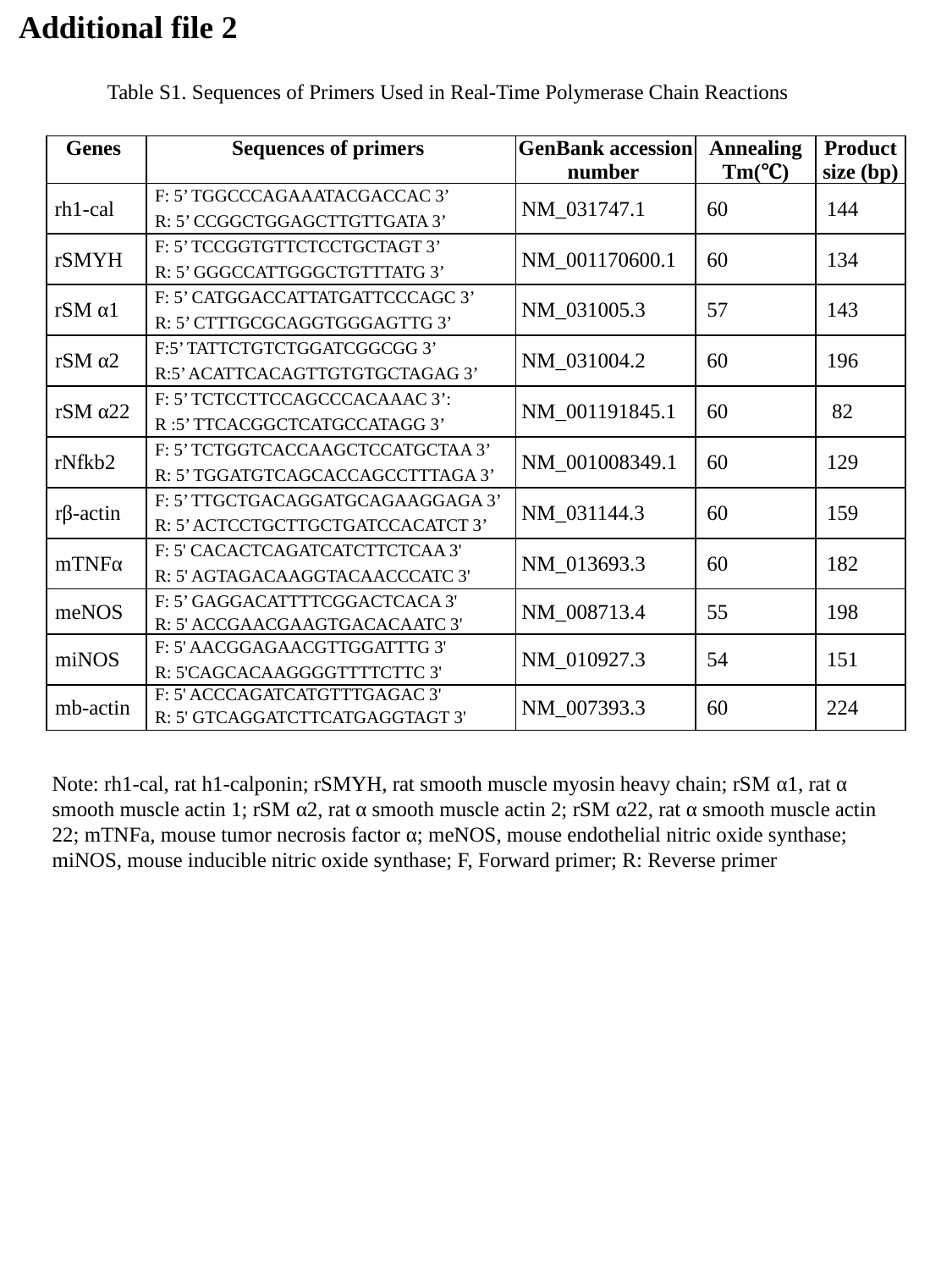

Additional file 2
 Table S1. Sequences of Primers Used in Real-Time Polymerase Chain Reactions
| Genes | Sequences of primers | GenBank accession number | Annealing Tm(℃) | Product size (bp) |
| --- | --- | --- | --- | --- |
| rh1-cal | F: 5’ TGGCCCAGAAATACGACCAC 3’ | NM\_031747.1 | 60 | 144 |
| | R: 5’ CCGGCTGGAGCTTGTTGATA 3’ | | | |
| rSMYH | F: 5’ TCCGGTGTTCTCCTGCTAGT 3’ | NM\_001170600.1 | 60 | 134 |
| | R: 5’ GGGCCATTGGGCTGTTTATG 3’ | | | |
| rSM α1 | F: 5’ CATGGACCATTATGATTCCCAGC 3’ | NM\_031005.3 | 57 | 143 |
| | R: 5’ CTTTGCGCAGGTGGGAGTTG 3’ | | | |
| rSM α2 | F:5’ TATTCTGTCTGGATCGGCGG 3’ | NM\_031004.2 | 60 | 196 |
| | R:5’ ACATTCACAGTTGTGTGCTAGAG 3’ | | | |
| rSM α22 | F: 5’ TCTCCTTCCAGCCCACAAAC 3’: | NM\_001191845.1 | 60 | 82 |
| | R :5’ TTCACGGCTCATGCCATAGG 3’ | | | |
| rNfkb2 | F: 5’ TCTGGTCACCAAGCTCCATGCTAA 3’ | NM\_001008349.1 | 60 | 129 |
| | R: 5’ TGGATGTCAGCACCAGCCTTTAGA 3’ | | | |
| rβ-actin | F: 5’ TTGCTGACAGGATGCAGAAGGAGA 3’ | NM\_031144.3 | 60 | 159 |
| | R: 5’ ACTCCTGCTTGCTGATCCACATCT 3’ | | | |
| mTNFα | F: 5' CACACTCAGATCATCTTCTCAA 3' | NM\_013693.3 | 60 | 182 |
| | R: 5' AGTAGACAAGGTACAACCCATC 3' | | | |
| meNOS | F: 5’ GAGGACATTTTCGGACTCACA 3' | NM\_008713.4 | 55 | 198 |
| | R: 5' ACCGAACGAAGTGACACAATC 3' | | | |
| miNOS | F: 5' AACGGAGAACGTTGGATTTG 3' | NM\_010927.3 | 54 | 151 |
| | R: 5'CAGCACAAGGGGTTTTCTTC 3' | | | |
| mb-actin | F: 5' ACCCAGATCATGTTTGAGAC 3' | NM\_007393.3 | 60 | 224 |
| | R: 5' GTCAGGATCTTCATGAGGTAGT 3' | | | |
Note: rh1-cal, rat h1-calponin; rSMYH, rat smooth muscle myosin heavy chain; rSM α1, rat α smooth muscle actin 1; rSM α2, rat α smooth muscle actin 2; rSM α22, rat α smooth muscle actin 22; mTNFa, mouse tumor necrosis factor α; meNOS, mouse endothelial nitric oxide synthase;
miNOS, mouse inducible nitric oxide synthase; F, Forward primer; R: Reverse primer
